# Anti-inflammatory effects of hyperbaric oxygen on irradiated laryngeal tissues^☆^

**DOI:** 10.1016/j.bjorl.2017.02.001

**Published:** 2017-02-27

**Authors:** Mitat Arıcıgil, Mehmet Akif Dündar, Abitter Yücel, Hamdi Arbağ, Abdullah Arslan, Meryem Aktan, Sıdıka Fındık, İbrahim Kılınç

**Affiliations:** a Necmettin Erbakan University, Department of Otorhinolaryngology Head and Neck Surgery, Konya, Turkey; b Horasan State Hospital, Department of Otorhinolaryngology Head and Neck Surgery, Erzurum, Turkey; c Necmettin Erbakan University, Department of Undersea and Hyperbaric Medicine, Konya, Turkey; d Necmettin Erbakan University, Department of Radiation Oncology, Konya, Turkey; e Necmettin Erbakan University, Department of Pathology, Konya, Turkey; f Necmettin Erbakan University, Department of Biochemistry, Konya, Turkey

**Keywords:** Hyperbaric oxygen, Neck radiotherapy, Inflammation, Oxigênio hiperbárico, Radioterapia de pescoço, Inflamação

## Abstract

**Introduction:**

To manage the complications of irradiation of head and neck tissue is a challenging issue for the otolaryngologist. Definitive treatment of these complications is still controversial. Recently, hyperbaric oxygen therapy is promising option for these complications.

**Objective:**

In this study, we used biochemical and histopathological methods to investigate the efficacy of hyperbaric oxygen against the inflammatory effects of radiotherapy in blood and laryngeal tissues when radiotherapy and hyperbaric oxygen are administered on the same day.

**Methods:**

Thirty-two Wistar Albino rats were divided into four groups. The control group was given no treatment, the hyperbaric oxygen group was given only hyperbaric oxygen therapy, the radiotherapy group was given only radiotherapy, and the radiotherapy plus hyperbaric oxygen group was given both treatments on the same day.

**Results:**

Histopathological and biochemical evaluations of specimens were performed. Serum tumor necrosis factor-α, interleukin-1β, and tissue inflammation levels were significantly higher in the radiotherapy group than in the radiotherapy plus hyperbaric oxygen group, whereas interleukin-10 was higher in the radiotherapy plus hyperbaric oxygen group.

**Conclusion:**

When radiotherapy and hyperbaric oxygen are administered on the same day, inflammatory cytokines and tissue inflammation can be reduced in an early period of radiation injury.

## Resumo

**Introdução:**

O manejo das complicações da irradiação do tecido da cabeça e pescoço é uma questão desafiadora para o otorrinolaringologista. O tratamento definitivo dessas complicações ainda é controverso. Recentemente, a oxigenoterapia hiperbárica tem sido uma opção promissora para estas complicações.

**Objetivo:**

Nesse estudo foram utilizados métodos bioquímicos e histopatológicos para investigar a eficácia do oxigênio hiperbárico contra os efeitos inflamatórios da radioterapia no sangue e nos tecidos laríngeos, quando a radioterapia e oxigênio hiperbárico são administrados no mesmo dia.

**Métodos:**

Trinta e dois ratos Wistar albinos foram divididos em quatro grupos. O grupo controle não recebeu tratamento, o grupo de oxigênio hiperbárico recebeu apenas oxigenoterapia hiperbárica, o grupo de radioterapia recebeu apenas radioterapia e o grupo de radioterapia com oxigênio hiperbárico recebeu ambos os tratamentos no mesmo dia.

**Resultados:**

Foram realizadas avaliações histopatológicas e bioquímicas dos espécimes. Os níveis séricos de fator de necrose tumoral-α, interleucina-1β e inflamação tecidual foram significativamente maiores no grupo de radioterapia do que no grupo de radioterapia mais oxigênio hiperbárico, enquanto que a interleucina-10 foi maior no grupo de radioterapia mais oxigênio hiperbárico.

**Conclusão:**

Quando a radioterapia e o oxigênio hiperbárico são administrados no mesmo dia, as citocinas inflamatórias e a inflamação tecidual podem ser reduzidas no período inicial da radiação.

## Introduction

Head and neck cancers are the most frequently occurring cancers worldwide. In epidemiologic studies, they are ranked 10th globally, and their incidence has increased considerably in the past 10 years. The management of head and neck cancer is complex and requires a multidisciplinary approach.^1^ Radiotherapy is used as a primary or adjuvant treatment option for the treatment of head and neck cancers. Irradiation of the neck region can be performed in the presence of neck lymph node metastasis from the larynx, or cancers of any other region, or in the various stages of laryngeal cancer.^2^ Apart from the therapeutic nature of radiotherapy, it also has definite hazardous effects on the surrounding tissues and these effects can occur at either early or late stages. Dry mouth, mucositis, Soft Tissue Radionecrosis (STRN), Osteoradionecrosis (ORN), and Laryngeal Radionecrosis (LRN) are some of the examples of these side effects.^2^, ^3^

Radiation causes the release of inflammatory mediators, marking the beginning of the inflammatory process, and these result in an increase in oxidizing activity in the damaged tissues.^4^ Radiation not only causes damage to tissues through Reactive Oxygen Species (ROS) and ionization, but also through the caspase pathway and cytochrome activation, and as a result, necrosis develops in the damaged tissues from ischemia and apoptosis.^5^, ^6^ The cell tries to combat this damaging effect by activating its defense system (anti-inflammatory cytokines, antioxidant activity).^7^

Recently, a number of scientific studies on the management of side effects of radiotherapy have been conducted, and antioxidant herbal medicine, various chemical agents, vitamins, exogenous antioxidant molecules, and Hyperbaric Oxygen (HBO_2_) have been used in these studies.^6^, ^8^, ^9^, ^10^, ^11^ HBO_2_ has been widely used for the treatment of various diseases for decades. Treatment of diabetic wounds, flap necrosis, and sudden hearing loss are some of the prominent examples.^12^, ^13^, ^14^

In this study, we used biochemical and histopathologic methods to investigate the efficacy of hyperbaric oxygen against the early inflammatory effects of radiotherapy on the laryngeal tissue when radiotherapy and HBO_2_ are administered on the same day.

## Methods

This study was reviewed and approved by the Institutional Animal Care and Use Committee and Local Instuitional Review Board. The number of rats in each group was restricted by the ethics committee.

### Experimental design

Thirty-two Wistar Albino rats were divided into four groups. The control group (*n* = 8) was given no treatment during the study period. The HBO_2_ treatment group (HBO_2_) (*n* = 8) was given only HBO_2_ throughout the study. The Radiotherapy (RT) group (*n* = 8) was given only RT throughout the study. The RT plus HBO_2_ group (RT + HBO_2)_ (*n* = 8) was given both RT and HBO_2_ on the same day.

### Animals

Wistar albino rats (female, 230 ± 20 g) were housed under standard conditions (20 ± 1 °C room temperature, 60 ± 10% humidity, and a 12/12 h light/dark cycle) in regular cages and allowed free access to food and water. All experiments were conducted in accordance with the Guide for the Care and Use of Laboratory Animals published by the US Public Health Service.

## Neck area irradiation

Radiotherapy was applied under general anesthesia with 10 mg/kg ketamine and 8 mg/kg xylazine intraperitoneally (i.p.) to immobilize the rats prior to radiation exposure. The animals were placed on a plexiglass tray and stabilized in the supine position. The neck region of each rat was defined by simulation and irradiated with 2 Gray (Gy) per minute, for a total dose of 18 Gy with 6 MV photon beams (linear accelerator, Siemens, Primus). The source-axis distant technique was used, and the distance from the source center to the larynx was 100 cm. A 10 mm bolus was applied above the neck to compensate for dose depth. Each rat was exposed to a single dose of radiation. The animals were returned to their home cages following irradiation.

### HBO_2_ treatment

The animals were treated with HBO_2_ in the research animals’ pressure room in the Animal Care and Research Unit. The rats were given HBO_2_ once a day, 6 days a week, for a period of 4 weeks. The rats were taken in their cages to the pressure room, and, after a 10 min 100% oxygen ventilation, the cabin pressure was regulated at 2.4 absolute atmosphere for 10 min. Ventilation was provided at intervals throughout the 90 min treatment. Exits from the pressure room took 10 min. The rats in the RT + HBO_2_ group were started on HBO_2_ treatment before radiotherapy on the same day that RT was given.

### Euthanasia protocol

Four weeks later, after the administration of RT, all the rats were placed under general anesthesia using 10 mg/kg ketamine and 8 mg/kg xylazine i.p. The rats’ hearts were punctured and blood withdrawn. Thereafter, they were immediately decapitated and their laryngeal tissues removed surgically.

### Histopathological and immunohistochemical evaluation

Twenty-four hours of tissue fixation in 10% formaldehyde was performed, and then, the tissues were embedded in paraffin blocks. The processed tissues were sliced in an microtome equipment. Five-micron-thick cross-sections from all of the blocks were printed onto lysine-coated slides. One cross-section from each block was taken for staining with hematoxylin and eosin (H + E); one cross-section was stained with immunohistochemical staining to show Leukocyte Common Antigen (LCA). Thermo AntiLCA Polyclonal Kit (PA5-23528, USA) was used to show LCA. All the preparations were analyzed with an Olympus BX51 light microscope by the same pathologist blinded to these groups. A general cartilage evaluation was performed using H + E staining, and the level of inflammation was evaluated using immunohistochemical LCA staining.

Inflammation and metaplasia levels were calculated as follows: 0–3. 0 – no inflammation, 1 – low inflammation, 2 – moderate inflammation, 3 – severe inflammation, 0 – no metaplasia, 1 – there is metaplasia.

### Biochemical evaluation

Measurement of proinflammatory and anti-inflammatory cytokines and oxidant values in rat plasma was conducted. The blood samples were transferred to K_3_-EDTA tubes and centrifuged at 1000 × *g* for 10 min at +4 °C. Plasma samples were separated and stored at −0 °C. The plasma samples were later defrosted for use. TNF α (Biolegend, San Diego, CA, USA), IL1-β and IL-10 (Boster Biological Technology, CA, USA) levels were measured in the homogenized samples by enzyme-linked immunosorbent assay (ELISA) using industrial kits as instructed by the manufacturers.

### Statistical analysis

Data were analyzed using the SPSS 23.0 (USA) packet program. For comparison within the groups, Kruskal–Wallis variance analysis was used. To determine significant differences between the groups, the Mann–Whitney *U* test with Bonferroni's correction was used. *p* < 0.05 was accepted as statistically significant.

## Results

### Histopathological and immunohistochemical finding

**Figure 1:**
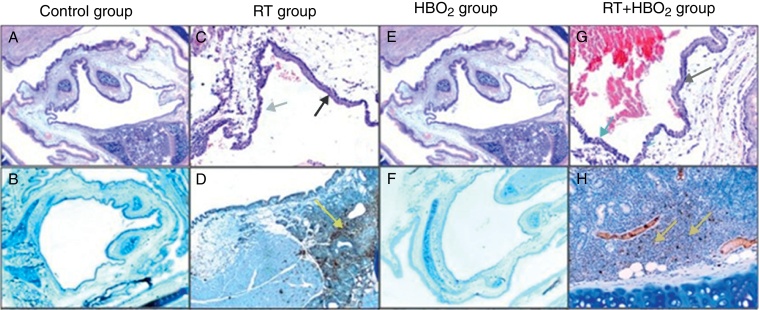
Representative photomicrographs of cartilage histology in the control, HBO_2_, RT and RT + HBO_2_ groups. Control group: (A) general appearance of H&E (×20); (B) Immunohistochemical LCA (×20). RT group: (C) H&E (×100) black arrow squamous metaplasia – blue arrow columnar epithelium. (D) Immunohistochemical LCA (×40) yellow arrow LCA positive lymphocytes. HBO_2_ group: (E) H&E (×20) general appearance; (F) Immunohistochemical LCA (×20). RT + HBO_2_ group: (G) H&E (×100) black arrows quamous metaplasia – blue arrow columnar epithelium; (H) Immunohistochemical LCA (×100) yellow arrow LCA positive lymphocytes.

Pathological variations and their distributions in the specimens in all the groups are shown in Table 1 and Fig. 1. Inflammation and metaplasia seen in laryngeal tissue were not statistically significant between the control and HBO_2_ groups (*p* > 0.05). The rate of inflammation and metaplasia in the cartilage tissue were significantly higher in the RT group compared to the control, HBO_2_, and RT + HBO_2_ groups (*p* < 0.05). The cartilage tissue of the rats in the RT + HBO_2_ group had a significantly higher inflammation and squamous metaplasia level than the control and HBO_2_ groups.

**Table 1:** Squamous metaplasia and inflammation levels of the rat groups.

Pathological changes	Control (*n* = 8)	HBO_2_ (*n* = 8)	RT (*n* = 8)	RT + HBO_2_ (*n* = 8)
*Squamous metaplasia*				
Metaplasia+	0	0	7	2
Metaplasia−	8	8	1	6

*Inflammation*				
No inflammation	8	7	0	0
Mild inflammation	0	1	1	4
Moderate inflammation	0	0	3	3
Severe inflammation	0	0	4	1

### Biochemical findings

Biochemical parameter results of all groups are shown in Table 2. There were no statistically significant differences in TNF-α, IL1-β, and IL-10 values between the control and HBO_2_ groups (*p* > 0.05). TNF-α and IL1-β values were significantly higher in the RT group compared to the control group (*p* < 0.05), whereas IL 10 was higher in the control group (*p* < 0.05). TNF α and IL1-β values were significantly higher in the RT group than in the RT + HBO_2_ group (*p* < 0.05), whereas IL-10 was higher in the RT + HBO_2_ group (*p* < 0.05). IL1-β values were significantly higher in the RT + HBO_2_ group than in the HBO_2_ group (*p* < 0.05), whereas IL-10 was higher in the HBO_2_ group (*p* < 0.05). There was no significant difference in TNF-α values between these two groups (*p* > 0.05). TNF-α and IL1-β were significantly higher in the RT + HBO_2_ group than in the control group (*p* < 0.05). There was no statistically significant difference in IL-10 between these two groups (*p* > 0.05) (Table 2, Table 3).

**Table 2:** Proinflammatory and anti-inflammatory cytokine values of the groups.

Groups	IL-1 β (mean/std dev) pg/ml	IL-10 (mean/std dev) pg/ml	TNF-α (mean/std dev) pg/ml
Control	22 ± 2.06	42 ± 6.8	17 ± 2.27
HBO_2_	23 ± 2.69	43 ± 6.7	19 ± 3.46
RT	55 ± 6.89	21 ± 1.89	31 ± 3.58
RT + HBO_2_	44 ± 2.85	32 ± 5.99	24 ± 2.62

**Table 3:** *p*-Values showing comparisons between the important groups.

Comparison of group	Control vs. HBO_2_ *p*-value	Control vs. RT *p*-value	Control vs. RT + HBO_2_ *p*-value	RT vs. RT + HBO_2_ *p*-value	HBO_2_ vs. RT *p*-value	HBO_2_ vs. RT + HBO_2_ *p*-value
IL-1β	0.462	0.006^a^	0.006^a^	0.03^a^	0.03^a^	0.006^a^
TNF-α	0.093	0.006^a^	0.006^a^	0.012^a^	0.006^a^	0.21
IL-10	0.754	0.006^a^	0.116	0.006^a^	0.006^a^	0.036^a^

a Statistically significant values.

## Discussion

Signs and symptoms after the completion of RT treatment were described as being early if they occurred within the first 3 months and late if they occurred after the first 6 months. The early and late effects of RT on the normal tissue are different with regard to both pathophysiology and clinical presentation. Changes related to the early effects of RT are either a result of increasing cell death from DNA injury or formation of ROS, whereas late changes related to RT are a result of vascular injury and fibrosis. These changes are usually progressive.^15^, ^16^, ^17^ In head and neck cancers, radiotherapy is administered in lower doses and in repeated sessions. The aim of administering a single high dose of radiotherapy in our study was to successfully induce radiation injury and the radiation dose that we administered was decided by considering previous similar studies.^7^ In our study, HBO_2_ treatment was started on the same day as radiotherapy because HBO_2_ was given for protective effect, not for the treatment of damaged tissue.

HBO_2_ is usually used to treat the late period radiation effects of radiotherapy. Narozny et al.^18^ reported that of 548 patients who were given RT to treat head and neck cancer, grade 3 and 4 chondroradionecrosis developed in six patients, and all had symptom remission after conventional treatment followed by HBO_2_. In another retrospective review, it was reported that none of five chondroradionecrosis patients treated with HBO_2_ required laryngectomy, and two of the four patients from this group who were dependent on tracheotomy were decanulated.^16^ Filntisis et al.^17^ administered adjuvant HBO_2_ treatment to 18 patients with grade 3 and 4 radionecrosis, and 13 (72.2%) of these patients showed major recovery after HBO_2_ treatment; 5 (27.8%) patients required a total laryngectomy due to unsuccessful treatment. Filntisis reported that HBO_2_ treatment is a safe and easy way to use adjuvant treatment in larynx radionecrosis that is resistant to conservative methods. Furthermore, in a study by Roh et al.,^19^ of six patients diagnosed with chondroradionecrosis and treated with early debridement and HBO_2_, recovery was seen in five patients, whereas one patient had to undergo a total laryngectomy due to failed treatment. In a study by Niezgoda et al.^20^ HBO_2_ was used in the treatment of various tissue injuries caused by radiation, and recovery was obtained in laryngoradionecrosis combined with a number of other tissue injuries.

In all the above-mentioned studies, HBO_2_ was given as treatment after injury had occurred, which mostly involved late radiation injury. In our study, HBO_2_ was given at the same time as radiotherapy to determine its effects on tissue inflammation the protection of tissue from inflammation. Consequently, it considerably reduced inflammation. When the rats were decapitated on the fourth week, inflammation that occurred due to radiotherapy was an early effect, and to some point, HBO_2_ had suppressed inflammation that could cause tissue injury. Six months must pass for the late effects to be realized.

Studies have researched the anti-inflammatory effect of HBO_2_. In a study by Chen et al.,^21^ the middle cerebellar artery of rats was clamped, rendering the brain hypoxic. HBO_2_ was given for treatment and was shown to decrease inflammatory cytokines and increase antioxidant activity. In our study, HBO_2_ decreased inflammatory cytokines and tissue inflammation but increased anti-inflammatory cytokine. In another study, colon injury was induced in rats by administering dextran sulfate sodium, and then HBO_2_ was given.^22^ HBO_2_ reduced pro inflammatory cytokines and suppressed inflammation. Arslan et al.^23^ induced acoustic trauma in the ear, treated it with dexamethasone and HBO_2_, and then compared these two agents in their study. They reported that when HBO_2_ is given, especially in the first 24 h, it can be as effective as dexamethasone as an anti-inflammatory agent. The anti-inflammatory effect of HBO_2_ is a common factor in these studies, and their results are similar to our present results.

Our study has some limitations. First, the early period anti-inflammatory effect of HBO_2_ against radiation injury was evaluated, but this anti-inflammatory effect on the late effects of radiation, such as soft tissue necrosis was not evaluated. Furthermore, the anti-inflammatory effect of HBO_2_ on the antitumor effects of radiotherapy is not known. The other limitation is that the effects of low and multiple radiation doses could not be predicted because a single large dose was given in this study.

## Conclusion

HBO_2_ plays an anti-inflammatory role in the early period after radiotherapy injury in tissue when given on the same day as radiotherapy. Further studies are needed to show the late effects when both agents are given on the same day as well as the effects on the tumoral tissue.

## Ethical considerations

The study has been conducted in the local Experimental Animals Research and Application Center of the Faculty of Medicine. The approval of the ethical board has been taken (number of approval of the ethics committee: 2015-008).

## Conflicts of interest

The authors declare no conflicts of interest.
